# Affective Network Neuroscience

**DOI:** 10.3389/fnins.2018.00895

**Published:** 2018-12-11

**Authors:** Sebastian Markett, Olga A. Wudarczyk, Bharat B. Biswal, Philippe Jawinski, Christian Montag

**Affiliations:** ^1^Department of Psychology, Humboldt University Berlin, Berlin, Germany; ^2^Department of Psychiatry & Psychotherapy, RWTH Aachen, Aachen, Germany; ^3^Department of Biomedical Engineering, New Jersey Institute of Technology, Newark, NJ, United States; ^4^MOE Key Lab for Neuroinformation, The Clinical Hospital of Chengdu Brain Science Institute, University of Electronic Science and Technology of China, Chengdu, China; ^5^Department of Molecular Psychology, Insitute of Psychology and Education, Ulm University, Ulm, Germany

**Keywords:** affective neurosience, connectome, network neuroscience, resting-state functional MRI, personality

The last years have seen the rise of a new paradigm in human neuroimaging: network neuroscience (Bassett and Sporns, 2017). Network neuroscience conceptualizes the brain as a connectome—an intricate network map of the brain where brain regions synchronize their activity via myriads of interconnecting nerve fibers. Network neuroscience is an interdisciplinary endeavor whose potential for cognitive science, the study of individual differences, and clinical research has been highlighted in several recent articles (Braun et al., 2015; Medaglia et al., 2015; Markett et al., 2018; Tompson et al., 2018). In the following, we will argue that network neuroscience provides an innovative toolbox that can also advance our understanding of affective processes in the brain, particularly when guided by (neuro)psychological theory.

The transient synchronization of activation between remote brain areas is typically interpreted as *functional connectivity* (Friston et al., 1993), while *structural connectivity* refers to white matter fiber tracts that connect between brain areas. Even though neuroimaging techniques for both types of brain connectivity have been available for over two decades (Biswal et al., 1995; Mori et al., 1999), it took two major developments in the mid-2000s to trigger the current enthusiasm for network neuroscience. The first new development was brought to the field by functional neuroimaging. By analyzing temporal synchronizations in the blood oxygen level dependent (BOLD) signal during stimulation-free resting state, it was shown independently by various groups that the brain is organized into large-scale functional networks that can be consistently identified across participants and time (Greicius et al., 2003; Beckmann et al., 2005; Damoiseaux et al., 2006). Brain areas that synchronize their activity at rest also tend to co-activate during task (Smith et al., 2009; Di et al., 2013), which has led to several systems neuroscience accounts of how functional networks might interact to support a wide range of behavioral and cognitive functions (Dosenbach et al., 2008; Menon, 2011). The second paradigm—based on structural neuroimaging at first—started out by demonstrating the feasibility of detailing brain connectivity in the form of a connectome map (Hagmann, 2005; Sporns et al., 2005). A connectome map can be inferred from imaging data by collating a parcellation scheme of the cortical ribbon with fiber tracking procedures applied to diffusion MRI. The resulting network map can be studied with tools from mathematical graph theory, in order to reveal the principles of network-level organization of brain connectivity (Bullmore and Sporns, 2009). The relationship between functional and structural connectivity is complex and often indirect (Mišić et al., 2016). But the current understanding is that structural connections represent a communication scaffold that enables transient functional couplings of brain regions into network modules that support a wide range of behavioral and cognitive functions (Park and Friston, 2013). Modern day connectomics therefore includes a structural and a functional branch that are ideally studied together. The approach is illustrated in Figure 1.

**Figure 1:**
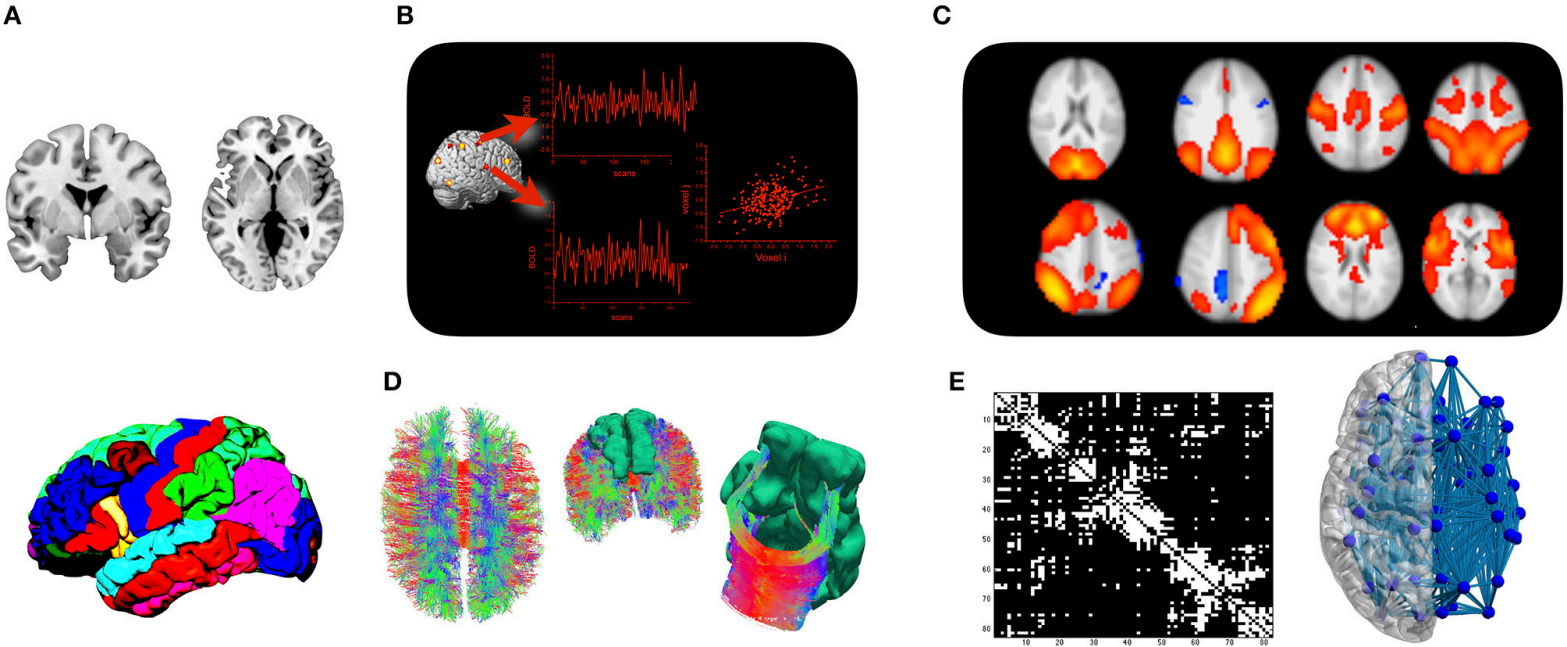
The connectome approach: **(A)** Cortical and subcortical gray matter are parcellated into a set of regions of interest. **(B)** Mean time courses of BOLD activity are extracted from each region of interest, and functional connectivity is assessed by analyzing statistical dependencies between any two regions. **(C)** Functional connectivity is organized into a set of large-scale networks at the brain level. **(D)** Fiber tracking is applied to diffusion MRI data to assess whether regions from the parcellation are structurally connected. **(E)** Results are either displayed in a connectivity matrix whose elements indicate whether two regions are connected or not, or displayed in a connectivity plot for anatomical reference.

The fact that the brain is a network, and that brain connectivity plays a crucial role in thought and behavior has been known since the early days of neuroscience. Previously, the study of structural and functional brain connectivity remained restricted to experimental animals, as the required methodology involved the injection of tracers or neurotoxins into brain tissue (Stephan, 2013). Due to the invasive nature, connectivity studies were often limited to single fiber tracts, and the assemblage of connectome maps was only possible when data were collated across many individual animals (Stephan, 2013). The current enthusiasm for network neuroscience based on non-invasive neuroimaging data reflects the fact that it allows cognitive and affective neuroscientists to catch up with connectivity analyses in human research participants. It also enables the holistic and repeated analysis of individual connectomes, particularly since it has been shown that macrolevel MRI-derived connectivity corresponds well with microlevel neuroarchitectonics (Scholtens et al., 2014). Network neuroscience represents first of all a new paradigmatic way of reasoning about the brain and second of all a fast-growing collection of methodological tools. Its full potential to the study of psychological phenotypes can be leveraged when its tools are applied to study brain connectivity in the context of psychological theory. In the following section, we will highlight the prospects of brain connectivity research in the context of three different influential theories on affect and emotion: The affective neuroscience theory (Panksepp, 1998), the reward sensitivity theory (Gilson et al., 2018), and the theory of constructed emotions (Barrett, 2017).

Affective neuroscience (AN) theory postulates seven primary emotional systems: SEEKING, LUST, CARE, PLAY on the side of positive emotions, and FEAR, ANGER/RAGE, and PANIC/SADNESS on the negative side (Panksepp, 1998, 2010; Montag and Panksepp, 2017a,b; Montag et al., 2017b; Davis and Montag, 2018). The distinct neural circuitry underlying the systems have been mainly mapped using localized electrical stimulation of the brains of experimental animals. For a detailed overview on the neuroanatomy underlying each primary emotional emotion see Panksepp (2011) and Montag and Panksepp (2016). Animals show behavioral responses consistent with basic emotions after stimulation of subcortical sites, such as the periaqueductal gray, the amygdala, or the medial forebrain bundle (Panksepp, 2010). As primary emotional systems, the seven circuits are thought to be innate and phylogenetically conserved across mammalian and non-mammalian species. An important topic for AN theory is therefore the translation of the animal data to humans. This endeavor is facilitated by the affective neuroscience personality scales (ANPS, Davis et al., 2003; Montag and Davis, 2018), a psychometric tool that has been developed on the background of AN theory and assesses individual differences in Pankseppian primary emotions. A straightforward application of tools from network neuroscience entails the mapping of connectivity patterns of subcortical structures implicated by electrical stimulation, followed by correlation analysis with ANPS scores. AN theory clearly argues for a localization of the phylogenetically old primary emotional systems in the brain's oldest layers (Panksepp et al., 2017). The validity of all network neuroscience approaches depends on the careful selection of seed regions for connectivity mapping (Fornito et al., 2013). The small subcortical structures with relevance for AN theory are particularly difficult to delineate. In our own work on the ANPS, we therefore made use of a cytoarchitectonic atlas to define seed regions in the amygdala sub-nuclei. This approach ensures a more accurate and anatomically informed perspective on the human amygdala (Roy et al., 2009; Eckstein et al., 2017). We found robust correlations between functional connectivity of the basolateral section of the amygdala to parietal cortices and SADNESS (Deris et al., 2017). This study was the first to address connectivity in human participants with respect to AN theory, and demonstrates the feasibility of this approach which is encouraging for further investigations.

Next to the study of individual differences with psychometric assessments of affective systems, it is crucial to use experimental approaches that aim at real behavior (Markett et al., 2014; Montag et al., 2017a). Several of such approaches have been proposed in the context of reward sensitivity theory (RST), a theory on approach and avoidance behavior (Gray and McNaughton, 2000). RST describes three systems in the brain that are thought to mediate between stimuli and response: the behavioral activation system (BAS) dealing with approach to appetitive stimuli, the fight-flight-freezing system (FFFS) dealing with active avoidance of threat, and the behavioral inhibition (BIS) system that mediates between the two in the case of response conflict, and deals with exploratory behavior in situations of uncertainty. RST does not resort to common language terms for emotions, but the operation of the FFFS can be equated with the emotion fear, while the operation of the BIS reflects anxiety. The dissociation between fear and anxiety is one of RST's hallmark features. The distance between a potential threat and the individual is thought to be decisive of whether the FFFS (proximal threat, fear) initiates a “get-me-out-of-here” reaction or the BIS (distant threat, anxiety) initiates a more careful assessment of the situation and strategic planning (Corr, 2013; Reuter et al., 2015). There are several behavioral assays for the study of the BIS and the FFFS: in a simulated runway-chase, participants operate a force-sensitive joystick to either escape or approach a virtual enemy (Perkins et al., 2009). Another approach includes a pac-man-style computer game where participants escape a virtual predator to avoid electric shocks (Mobbs et al., 2007). Distance to threat has been shown to map on a functional gradient in brain response, where proximal threat activates subcortical regions, such as periaqueductal gray and the (central nuclei of the) amygdala, and activation foci shift along a functional axis toward ventromedial prefrontal cortex with increasing distance to the threat (together with activation of the lateral amygdala). The defensive-distance gradient in the brain suggests an underlying network with information exchange along the functional axis. This, however, has not been formerly addressed as of yet. Network neuroscience offers tools to study modulations of functional connectivity by task context (Gerchen et al., 2014), including its dynamic changes over time (Muldoon and Bassett, 2016), and the directionality of information transfer (Gilson et al., 2018). The application of these methods with regard to predictions from RST represent excellent examples where the combination of network neuroscience and psychological theory may advance our understanding of affective systems in the human brain.

A more recent theory on affect and emotion stands as antithesis to previous accounts on primary emotions. The theory of constructed emotions (TCE, Barrett, 2017) represents a departure from the common neo-behavioristic paradigm in psychology, by moving the spotlight away from stimuli and neural systems that mediate between stimulus and response. The theory of constructed emotions follows a recent line of argumentation that the brain uses its past experience to engage in predictive modeling of the environment (Raichle, 2010). According to this perspective, emotions are constructed by the brain when it uses its model of the environment to make sense of incoming information (Barrett, 2006). TCE is quite radical in its opposition to previous accounts which has resulted in severe criticism (Panksepp, 2007). But TCE makes interesting statements on brain networks that are worth exploring. Functional connectivity mapping, for instance, has failed to delineate clear boundaries between functional systems associated with several primary emotions (Touroutoglou et al., 2015), a finding corroborated by evidence from multivariate pattern analyses (Clark-Polner et al., 2017). Functional connectivity systems for different emotions seem to converge within the insula-opercular network, a network that has been implicated in the detection of saliency (Seeley et al., 2007). TCE assumes a central role of the insula-opercular network in the conceptualization of emotions by tuning the brain's internal model of the environment to sensory signals (Barrett, 2017). Through this, TCE provides a theoretical account for other findings that have implicated the salience network in individual differences in the sensitivity to anxiety and negative affect (Markett et al., 2013, 2016). At present it is unclear, whether the TCE account can be unified with the older theories on primary emotional systems. In theory it should be possible, because primary emotional systems seem to operate at the bottom of our minds, whereas constructivist highlight neocortical processes (Panksepp, 2010).

The new field of network neuroscience with its fast growing methodological toolbox can make valuable contributions in advancing current theoretical accounts on affect and emotion. We wish to encourage further research into this direction, as well as efforts toward an *affective network neuroscience*. As any new field of study, network neuroscience is currently facing rapid methodical developments. These aim at the core challenges of the paradigmatic conceptualization of the brain as a network, such as more accurate parcellations for the cortical ribbon or better ways to measure functional connectivity, including its dynamics and directedness. Studying affect and emotion in terms of information transfer between interacting brain regions will hopefully lead to an algorithmic understanding of affective processing in the brain. This will have exciting prospects for other branches of neuroscience, e.g., for neuropsychopharmacology and molecular neurogenetics. It will also be an important step toward better treatment options for affective disorders (Richter et al., 2017) that constitute a significant public health burden with negative impact to those afflicted (Wittchen et al., 2011; Montag et al., 2017a).

## Author Contributions

SM and CM conceptualized the paper. OW, BB, and PJ provided critical points and revision. SM drafted the manuscript. OW, BB, PJ, and CM revised the manuscript.

### Conflict of Interest Statement

The authors declare that the research was conducted in the absence of any commercial or financial relationships that could be construed as a potential conflict of interest.
